# Progressive Axonal Degeneration of Nigrostriatal Dopaminergic Neurons in Calcium-Independent Phospholipase A_2_β Knockout Mice

**DOI:** 10.1371/journal.pone.0153789

**Published:** 2016-04-14

**Authors:** Goichi Beck, Koei Shinzawa, Hideki Hayakawa, Kousuke Baba, Hisae Sumi-Akamaru, Yoshihide Tsujimoto, Hideki Mochizuki

**Affiliations:** 1 Department of Neurology, Osaka University Graduate School of Medicine, Suita, Osaka, Japan; 2 Department of Medical Genetics, Osaka University Graduate School of Medicine, Suita, Osaka, Japan; 3 Osaka Medical Center for Cancer and Cardiovascular Diseases, Osaka, Japan; Hudson Institute, AUSTRALIA

## Abstract

Calcium-independent phospholipase A_2_β (iPLA_2_β, *PLA2G6*) is essential for the remodeling of membrane glycerophospholipids. Mutations in this gene are responsible for autosomal recessive, young onset, L-dopa-responsive parkinsonism (PARK14), suggesting a neurodegenerative condition in the nigrostriatal dopaminergic system in patients with *PLA2G6* mutations. We previously observed slowly progressive motor deficits in iPLA_2_β-knockout (KO) mice. To clarify whether a deficiency of iPLA_2_β leads to the degeneration of nigrostriatal dopaminergic neurons, we analyzed the striatum of iPLA_2_β-KO mice. At all clinical stages, nerve terminals in the striatum were immunopositive for tyrosine hydroxylase (TH) and dopamine transporter (DAT) in wild-type (WT) control mice. In iPLA_2_β-KO mice, focal loss of nerve terminals positive for TH and DAT was found from 56 weeks (early clinical stage), although iPLA_2_β-KO mice at 56 weeks showed no significant decrease in the number of dopaminergic neurons in the substantia nigra compared with age-matched WT mice, as reported previously. At 100 weeks (late clinical stage), greater decreases in DAT immunoreactivity were observed in the striatum of iPLA_2_β-KO mice. Moreover, strongly TH-positive structures, presumed to be deformed axons, were observed in the neuropils of the striatum of iPLA_2_β-KO mice starting at 15 weeks (preclinical stage) and increased with age. These results suggest that the degeneration of dopaminergic neurons occurs mainly in the distal region of axons in iPLA_2_β-KO mice.

## Introduction

Calcium-independent phospholipase A_2_β (iPLA_2_β) is a phospholipase A_2_ family member that hydrolyzes the *sn-2* ester bond in phospholipids including glycerophospholipids, such as phosphatidylcholine (PC), to yield free fatty acids and lysophospholipids [1]. iPLA_2_β, encoded by the *PLA2G6* gene, has several functions including membrane phospholipid remodeling [2], fatty acid oxidation [3], release of docosahexaenoic acid (DHA) and arachidonic acid (AA) [4], cell growth and signaling [5], and cell death [6]. In particular, iPLA_2_β is considered to be critical in cell membrane homeostasis [1]. PC levels, which are abundant in mammalian cell membranes and are key in maintaining membrane integrity, are regulated by the opposing actions of iPLA_2_β and cytidylylphosphocholine transferase [7].

In 2006, mutations in the *PLA2G6* gene were identified in an autosomal recessive neurodegenerative disease classified as infantile neuroaxonal dystrophy (INAD) and in neurodegeneration brain iron accumulation (NBIA type 2) [8]. In 2008, iPLA_2_β-knockout (KO) mice were reported to show progressive motor deficits, with neuropathological changes very similar to those of INAD [9, 10]. Defects in iPLA_2_β lead to a relative abundance of membrane PC, particularly PC with DHA, and to secondary structural abnormalities in the presynaptic membranes of axon terminals [11]. These abnormalities may underlie the axonal pathology observed in INAD, including the presence of tubulovesicular structures [12].

In 2009, *PLA2G6* was reported as the gene responsible for another autosomal recessive neurodegenerative disease, early- and adult-onset dystonia-parkinsonism (PARK14) [13]. To date, several mutations in the *PLA2G6* gene have been reported to cause PARK14 [14, 15, 16, 17, 18]. The main clinical features of PARK14 are extrapyramidal symptoms such as tremor, bradykinesia, rigidity, and generalized dystonia. These symptoms are responsive to L-dopa (L-3,4-dihydroxyphenylalanine) [13, 15, 16], suggesting that the nigrostriatal dopaminergic system is impaired to some extent in patients with *PLA2G6* mutations.

Tyrosine hydroxylase (TH) catalyzes the conversion of the amino acid L-tyrosine to L-Dopa, a dopamine precursor. After synthesis, dopamine is transported from the cytosol into synaptic vesicles by vesicular monoamine transporter 2 (VMAT2). Dopamine binds to and activates dopamine receptors in the synapse, and is quickly released from their receptors after an action potential. Then, they are absorbed back into the presynaptic cell via reuptake mediated by the dopamine transporter (DAT). Once back in the cytosol, dopamine is repackaged into vesicles by VMAT2, making it available for future release [19, 20, 21].

We previously demonstrated that insufficient remodeling of both the mitochondrial inner membrane and presynaptic membrane cause axonal degeneration in spinal cords and peripheral nerves of iPLA_2_β-KO mice [11]. The present study investigates how depletion of iPLA_2_β affects the nigrostriatal dopaminergic nervous system. We counted the numbers of TH- and Nissl-double-positive cells in the substantia nigra pars compacta (SNpc) in a blind manner in iPLA_2_β-KO mice and WT mice, and showed that no evidence of dopaminergic cell loss was observed in iPLA_2_β-KO mice before 56 weeks of age (early clinical stage) in the previous report [22]. Here, we performed neuropathological analyses of the striatum in iPLA_2_β-KO mice.

## Materials and Methods

### Animals

This study used mice with homozygous disruption of the iPLA_2_β gene on a C57BL/6 background [10] aged 15 weeks (n = 3, pre-clinical stage, 1 male and 2 females), 56 weeks (n = 7; early clinical stage; 3 males and 4 females), and 100 weeks (n = 8; late clinical stage; 5 males and 3 females) and wild-type (WT) control mice aged 15 weeks (n = 3; all males), 56 weeks (n = 6; all males), and 100 weeks (n = 8; 4 males and 4 females). After administration of an overdose of isoflurane, each animal was perfused with phosphate-buffered saline (PBS) and then 4% paraformaldehyde, followed by removal of the brain. Brains were immersed in the same fixative overnight at 4°C and then dehydrated and embedded in paraffin blocks. Paraffin sections (thickness, 4 μm) were prepared for immunohistochemistry. For free-floating immunohistochemistry, hemisphere brain blocks of mice were fixed overnight in 4% paraformaldehyde in PBS and then immersed in PBS containing 30% sucrose until sinking. Coronal sections of the brains were cut serially at 20-μm thickness using a cryostat (CM1850; Leica Microsystems).

This study was carried out in strict accordance with the Guidelines for Animal Experimentation of the Japanese Association for Laboratory Animal Science. All animals were handled in accordance with the Guidelines for Animal Experimentation of Osaka University. The experimental protocol was approved by the Ethical Review Committee for Animal Experimentation of Osaka University School of Medicine (Permit Number: 26-044-000). All efforts were made to minimize suffering.

### Immunohistochemistry

Brain sections of iPLA_2_β-KO mice aged 15 weeks (n = 3, 1 male and 2 females), 56 weeks (n = 4, 4 females), and 100 weeks (n = 5, 2 males and 3 females), and WT mice aged 15 weeks (n = 3, all males), 56 weeks (n = 3, all males), and 100 weeks (n = 5, 1 male and 4 females) were used.

Deparaffinized sections were incubated for 30 min in 0.3% H_2_O_2_ to quench endogenous peroxidase activity, and then washed with PBS. The primary antibodies used were rabbit polyclonal antibodies against TH (1:500, Calbiochem), VMAT2 (1:500, Novus Biologicals), DAT (1:500, Novus Biologicals), and microtubule-associated protein 2 (neuron-specific cytoskeletal proteins that are enriched in dendrites, MAP2, 1:1000, Sigma-Aldrich), and mouse monoclonal antibodies against phosphorylated neurofilament (SMI31, 1:10000, Sternberger Monoclonals) and ubiquitin (1:500, Novus Biologicals).

Goat anti-rabbit and anti-mouse immunoglobulins conjugated to peroxidase-labeled dextran polymer (Dako Envision+, Dako Corp.) were used as secondary antibodies. Reaction products were visualized with 3,3'-diaminobenzidine tetrahydrochloride (DAB, Vector Laboratories), and hematoxylin was used to counterstain the cell nuclei. Autoclave treatment was performed for 9 min before incubation with the antibody against DAT.

For double immunohistochemistry, secondary antibodies were visualized with the Vectastain kit (Vector Laboratories). Double staining was carried out by staining first with DAB (brown) followed by Vectastain (blue).

### Free-Floating Immunohistochemistry

Brain tissues of 56-week-old iPLA_2_β-KO mice (n = 3, all males) and WT mice (n = 3, all males) were used. Free-floating sections were washed in PBS medium containing 0.05% Triton X-100 (PBS-T) and then incubated for 30 min with 0.3% H_2_O_2_ to quench endogenous peroxidase activity. The sections were soaked with blocking agents and then incubated with primary antibodies dissolved in dilution reagent at 4°C for 24 h. The Vector M.O.M. Immunodetection Kit (Vector Laboratories) was used for blocking and antibody dilution according to the manufacturer’s instructions. A rabbit polyclonal antibody against TH (1:1000, Calbiochem) was used as the primary antibody. Goat anti-rabbit immunoglobulin conjugated to peroxidase-labeled dextran polymer (Dako Envision+, Dako Corp.) was used as the secondary antibody. Reaction products were visualized using DAB (Vector Laboratories). The sections were mounted on glass slides and counterstained with cresyl violet (Nissl staining).

### Assessment of Terminal Densities in the Striatum

Brain sections of iPLA_2_β-KO mice aged 15 weeks (n = 3, 1 male and 2 females), 56 weeks (n = 4, 4 females), and 100 weeks (n = 5, 2 males and 3 females), and WT mice aged 15 weeks (n = 3, all males), 56 weeks (n = 3, all males), and 100 weeks (n = 5, 1 male and 4 females) were used. For the analysis of optical densities of nerve terminals positive for VMAT2, brain tissues of 56-week-old iPLA_2_β-KO mice (n = 4, all females) and WT mice (n = 3, all males) were used.

The relative optical densities of TH-, DAT- and VMAT2-immunopositive fibers in the dorsal striatum were quantified using computer-assisted image analysis techniques (Image J, National Institutes of Health) [23]. For each mouse, 3–5 paraffin sections were used and 10–13 images were captured in black and white 8-bit monochrome using a digital camera attached to a microscope (oil immersion, 100× objective). Digital images were captured using the same exposure settings for all experimental cases. The optical density of the striatum of 15-week-old WT mice stained with hematoxylin was subtracted as background. The decrease in TH- and DAT-immunoreactivities was determined as the percent loss in iPLA_2_β-KO mice at all ages and WT mice aged 56 weeks and 100 weeks compared with the value for WT mice aged 15 weeks. The decrease in optical densities of VMAT2-positive fibers was determined as the percent loss in iPLA_2_β-KO mice at 56 weeks compared with the value for age-matched WT mice.

### Quantitative Pathological Analysis

To study the distal regions of axons of nigro-striatal dopaminergic neurons, TH-stained paraffin sections of the striatum of iPLA_2_β-KO mice aged 15 weeks (n = 3, 1 male and 2 females), 56 weeks (n = 4, 4 females), and 100 weeks (n = 5, 2 males and 3 females) were used. 3–5 paraffin sections from each KO mouse were examined. Video images were obtained using a digital camera connected to a microscope (40 × objective). The numbers of strongly TH-positive round structures exceeding 3 μm in diameter were counted in each mouse. The diameters of round structures were measured with the aid of image analysis software (VH-H1A5, Keyence), and the data were compared between iPLA_2_β-KO mice aged 15, 56, and 100 weeks.

### Western Blotting

Brain tissues of iPLA_2_β-KO mice aged 100 weeks (n = 3, all males) and WT mice aged 100 weeks (n = 3, all males) were used. Tissue preparation was done using the method described in our previous report [22]. Protein concentrations were determined by Lowry method. Proteins (10 μg for each mouse) were separated on 15% sodium dodecyl sulfate polyacrylamide gel electrophoresis (SDS-PAGE), electrotransferred to a polyvinylidene difluoride (PVDF) membrane (Bio-Rad), blocked with 5% nonfat milk in TBS–Tween buffer for 60 min at room temperature, and incubated overnight at 4°C with the primary antibodies. The primary antibodies used were a rabbit polyclonal antibody against TH (1:1000, Abcam), a rabbit polyclonal antibody against DAT (1:500, Novus Biologicals), and a mouse monoclonal antibody against glyceraldehyde-3-phosphate dehydrogenase (GAPDH, Millipore, 1:1000). The membrane was washed 3 times with TBS–Tween buffer for 30 min (10 min per wash), and then incubated for 60 min in horseradish peroxidase-conjugated goat anti-mouse or anti-rabbit IgG at room temperature. After extensive washing, the bands were visualized with enhanced chemiluminescence's reagents (ECL Prime Western Blotting Detection System, GE Healthcare) and exposed to X-ray film. The densitometry of the bands was quantified using computer-assisted image analysis techniques (Image J, National Institutes of Health).

### Statistical Analysis

The Wilcoxon’s rank sum test was used for two groups comparisons (EXCEL Toukei ver. 6.0, ESUMI). A probability of p < 0.05 was taken to indicate statistical significance.

## Results

### Immunohistochemical Analysis of Dopaminergic Nerve Markers

**Fig 1:**
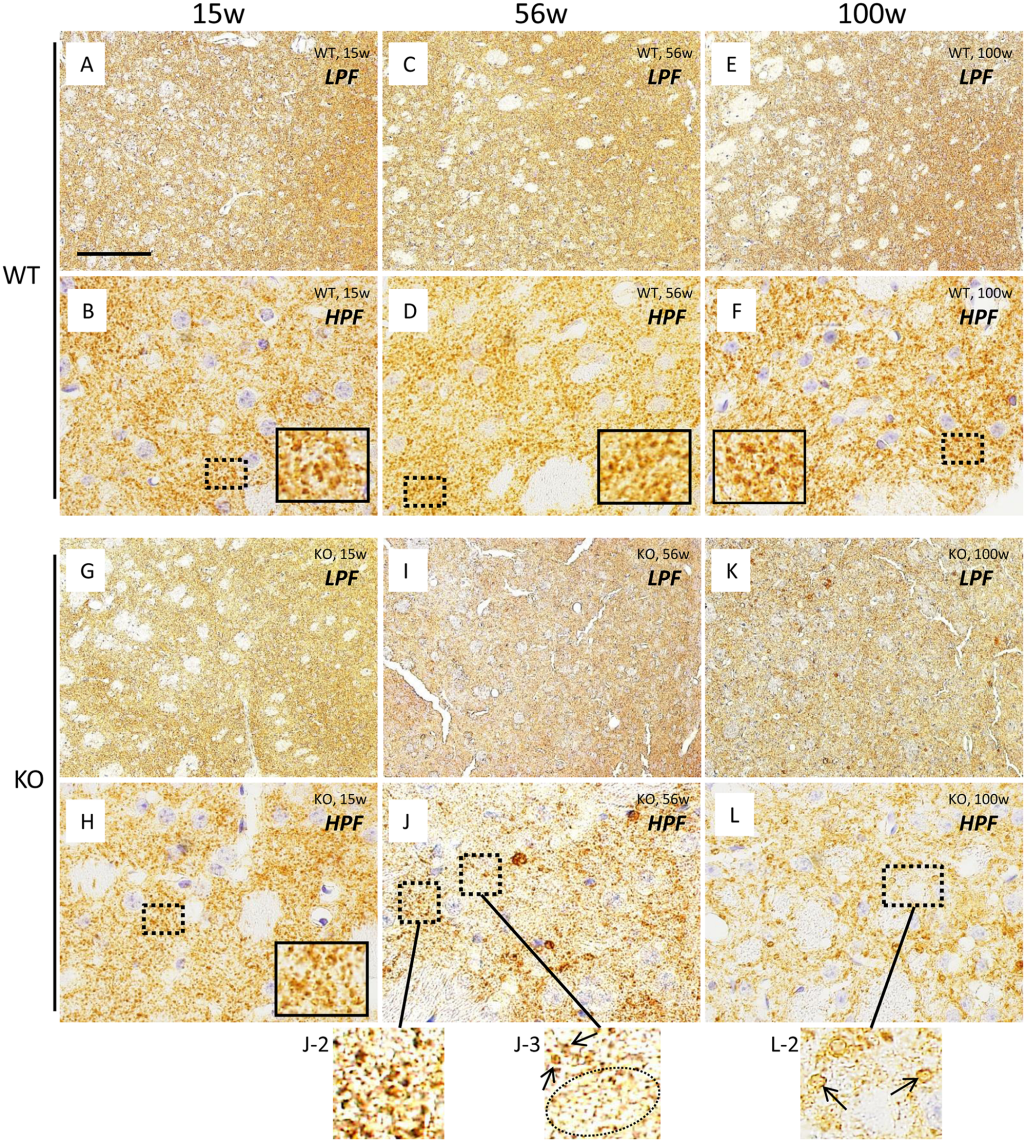
Immunohistochemical analysis for TH in the striatum of WT control mice and iPLA_2_β-KO mice. Representative photographs of the striatum immunostained with TH in WT mice at 15 weeks (A, B), 56 weeks (C, D), and 100 weeks (E, F) and iPLA_2_β-KO mice at 15 weeks (G, H), 56 weeks (I, J), and 100 weeks (K, L). (A), (C), (E), (G), (I), and (K) are low power fields (LPF) and (B), (D), (F), (H), (J), (L) are high power fields (HPF). (A-F): In the low power fields, neuropils of the striatum are diffusely stained with TH in WT mice at 15 weeks (A), 56 weeks (C), and 100 weeks (E). In the high power fields, many nerve fibers strongly immunopositive for TH were observed in neuropils of WT mice at 15 weeks (B), 56 weeks (D), and 100 weeks (F). The insets in (B), (D), (F), and (H) are high magnifications of the dotted square from their respective panel. (G-L): In the low power fields, neuropils of the striatum are diffusely stained with TH in iPLA_2_β-KO mice at 15 weeks (G), 56 weeks (I), and 100 weeks (K). In the high power fields, many fibers positive for TH were observed in neuropils of the striatum in iPLA_2_β-KO mice at 15 weeks (H), which are almost equal in number to those of WT mice at 15 weeks (B). In iPLA_2_β-KO mice at 56 weeks, focal loss of TH-positive fibers is seen in some areas (dotted circle in J-3), while the density of TH-positive fibers (arrows in J-3) is preserved in other areas (J-2, 3). In iPLA_2_β-KO mice at 100 weeks, the density of TH-positive fibers (arrows in L-2) are lower than that of WT mice at 100 weeks (F) and iPLA_2_β-KO mice at 56 weeks (J-2, 3). Panels (J-2), (J-3), and (L-2) are high magnifications of the dotted squares in (J) and (L), respectively. Scale bar in (A) represents 100 μm in (A), (C), (E), (G), (I), and (K), and 25 μm in (B), (D), (F), (H), (J), and (L).

**Fig 2:**
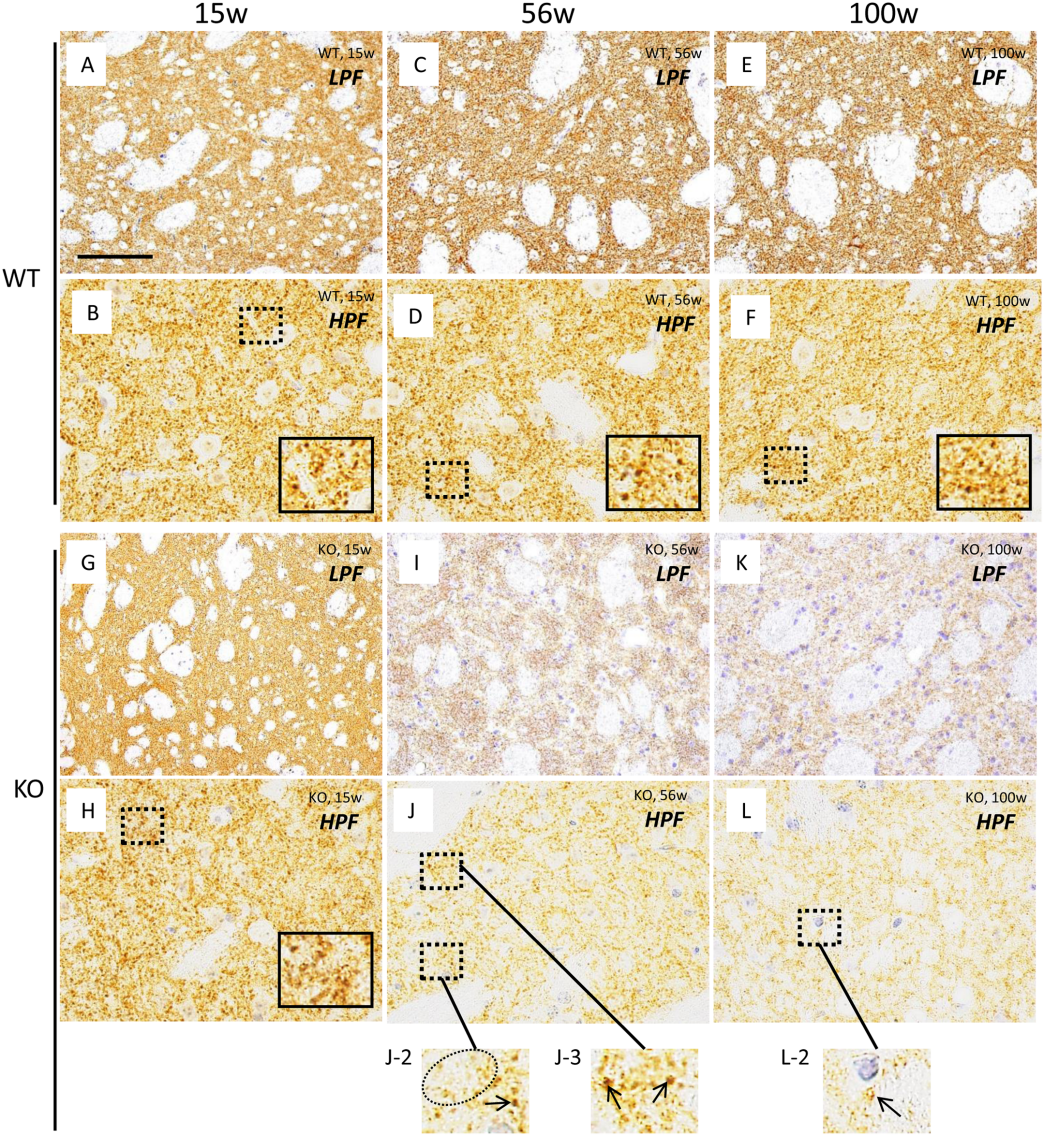
Immunohistochemical analysis of DAT in the striatum of WT control mice and iPLA_2_β-KO mice. Representative photographs of the striatum immunostained with DAT in WT mice at 15 weeks (A, B), 56 weeks (C, D), and 100 weeks (E, F) and iPLA_2_β-KO mice at 15 weeks (G, H), 56 weeks (I, J), and 100 weeks (K, L). (A), (C), (E), (G), (I), and (K) are low power fields (LPF), and (B), (D), (F), (H), (J), and (L) are high power fields (HPF). (A-F): In the low power fields, neuropils of the striatum were seen diffusely stained with DAT in WT mice at 15 weeks (A), 56 weeks (C), and 100 weeks (E). In the high power fields, many nerve fibers strongly immunostained with DAT were observed in neuropils of WT mice at 15 weeks (B), 56 weeks (D), and 100 weeks (F). The insets in (B), (D), (F), and (H) are high magnifications of the dotted squares from their respective panel. (G-L): In the low power fields, neuropils of the striatum were seen diffusely stained with DAT in iPLA_2_β-KO mice at 15 weeks (G). In iPLA_2_β-KO mice at 56 weeks (I) and at 100 weeks (K), neuropils are immunostained with DAT more weakly than those of age-matched WT mice (C, E) and iPLA_2_β-KO mice at 15 weeks (G). In the high power fields, many fibers strongly positive for DAT were observed in neuropils of the striatum of iPLA_2_β-KO mice at 15 weeks (H), nearly equal in numbers to those of WT mice at 15 weeks (B). In iPLA_2_β-KO mice at 56 weeks, focal loss of DAT-positive fibers is seen in some areas (dotted circle in J-2), while the density of TH-positive fibers (arrows in J-2 and J-3) is preserved in other areas (J-2, J-3). In iPLA_2_β-KO mice at 100 weeks, only a few fibers weakly positive for DAT (an arrow in L-2) were seen. Panels (J-2), (J-3), and (L-2) are high magnifications of the dotted squares in (J) and (L), respectively. Scale bars in (A), (C), (E), (G), (I), and (K) represent 100 μm and 25 μm in (B), (D), (F), (H), (J), and (L).

Dopaminergic innervation in the striatum was studied by immunohistochemical localization of TH and DAT. In WT mice at all ages (15, 56, and 100 weeks), striatal neuropils were diffusely immunostained with the two dopaminergic presynaptic markers (TH and DAT) (Figs 1A–1F and 2A–2F). In the striatum of WT mice of all ages, high magnification revealed many dots strongly positive for TH (Fig 1B, 1D and 1F) and DAT (Fig 2B, 2D and 2F), representing the distal regions of axons of dopaminergic neurons.

In iPLA_2_β-KO mice at 15 weeks (*pre-clinical stage*), neuropils of the striatum were diffusely immunopositive for TH (Fig 1G) and DAT (Fig 2G) in the low power fields. In the high power fields, there were many axons strongly positive for TH (Fig 1H) and DAT (Fig 2H), equal in numbers to those of WT mice at 15 weeks (Figs 1B and 2B). In iPLA_2_β-KO mice at 56 weeks (*early clinical stage*) and 100 weeks (*late clinical stage*), neuropils of the striatum were diffusely immunopositive for TH (Fig 1I and 1K) in the low power fields. However, in the high power fields, focal loss of fibers positive for TH was observed in some areas of the striatum in iPLA_2_β-KO mice at 56 weeks (Fig 1J); this finding was not seen in WT mice at 56 weeks (Fig 1D) or iPLA_2_β-KO mice at 15 weeks (Fig 1H). Moreover, in the striatum of iPLA_2_β-KO mice at 100 weeks, the number of fibers positive for TH was significantly lower in the high power fields (Fig 1L) compared with those of WT mice at 100 weeks (Fig 1F) and iPLA_2_β-KO mice at 56 weeks (Fig 1J).

In iPLA_2_β-KO mice at 56 weeks (Fig 2I) and 100 weeks (Fig 2K), the immunoreactivities for DAT in the striatum were prominently weak in comparison with those of WT mice at 56 weeks (Fig 2C) and 100 weeks (Fig 2E) in the low power fields. In the high power fields, the numbers of axons positive for DAT were significantly lower, and focal loss of DAT-positive fibers was higher in neuropils of the striatum of iPLA_2_β-KO mice at 56 weeks (Fig 2J) compared with those of WT mice at 56 weeks (Fig 2D) and iPLA_2_β-KO mice at 15 weeks (Fig 2H). Moreover, only a few axons were weakly positive for DAT in iPLA_2_β-KO mice at 100 weeks (Fig 2L).

**Fig 3:**
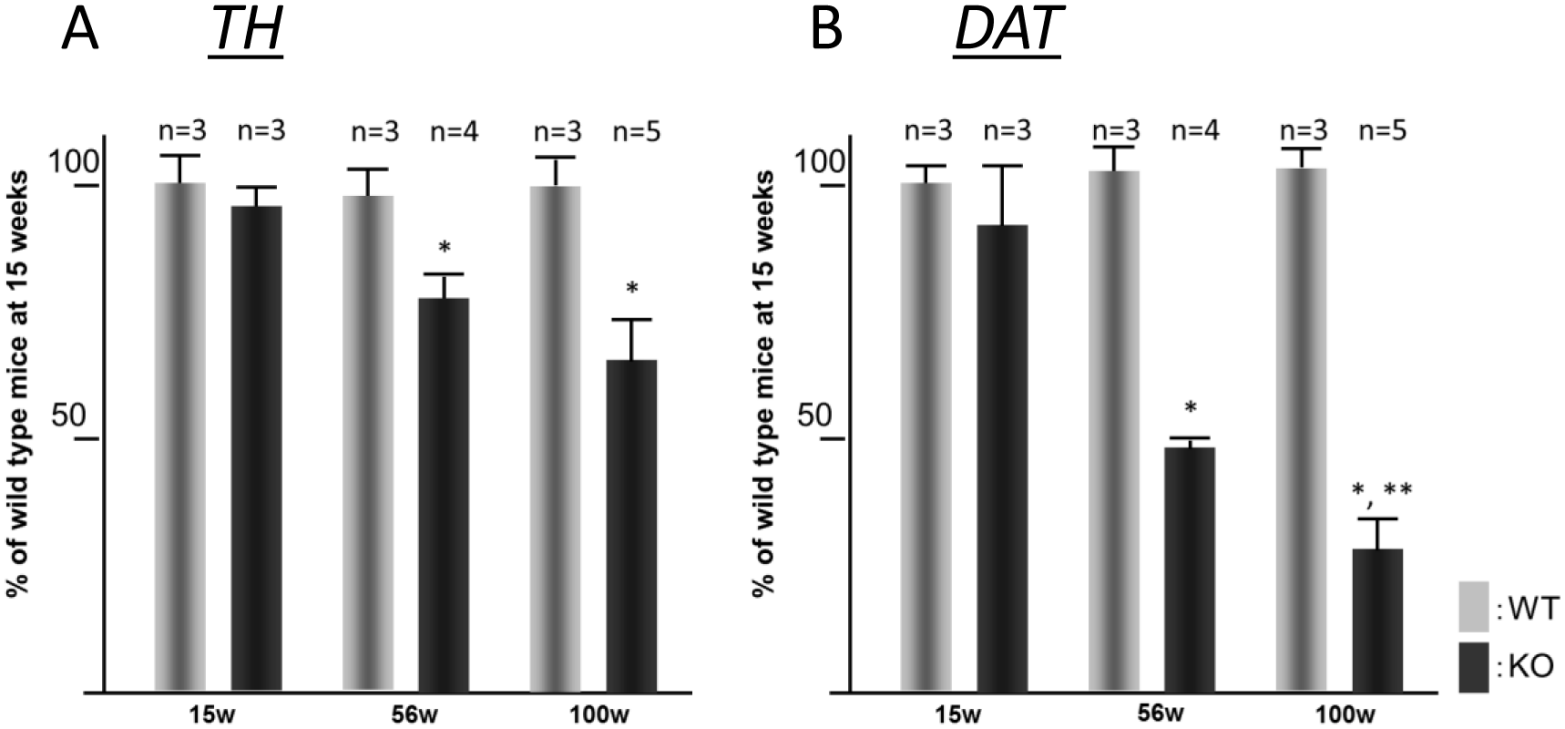
Quantitative analysis of optical densities of TH and DAT in the striatum. Histograms show quantitative analysis of optical densities of TH (A) and DAT (B) immunostaining in the striatum. WT mice, gray bars; KO mice, black bars. Data are presented as the mean ± standard deviation. The number (n) of animals examined is indicated in each histogram. Vertical axis in both (A) and (B) shows percent density relative to WT mice at 15 weeks. (A) Symbols indicate statistically significant differences; *p < 0.05 vs. age-matched WT mice and iPLA_2_β-KO mice at 15 weeks (Wilcoxon’s rank sum test). (B) Symbols indicate statistically significant differences; *p < 0.05 vs. age-matched WT mice and iPLA_2_β-KO mice at 15 weeks (Wilcoxon’s rank sum test) and **p < 0.05 vs. iPLA_2_β-KO mice at 56 weeks (Wilcoxon’s rank sum test).

By densitometric analysis, no significant differences in the immunoreactivity levels for the dopaminergic presynaptic markers (TH and DAT) were observed in WT mice between the three ages (Fig 3A and 3B). No significant difference was found in the optical density of either TH or DAT between iPLA_2_β-KO mice at 15 weeks and age-matched WT mice (Fig 3A and 3B). However, in iPLA_2_β-KO mice at 56 weeks, there was a statistically significant 20%–30% decrease in the optical density of TH compared with that of WT mice at 56 weeks and iPLA_2_β-KO mice at 15 weeks (*p* < 0.05, Wilcoxon’s rank sum test) (Fig 3A). The level of immunoreactivity for TH was lower in iPLA_2_β-KO mice at 100 weeks than in iPLA_2_β-KO mice at 56 weeks, although statistical significance was not observed (*p* > 0.05, Wilcoxon’s rank sum test) (Fig 3A). In iPLA_2_β-KO mice at 56 weeks, there was a significant decrease in the optical density of DAT compared with that of WT mice at 56 weeks and iPLA_2_β-KO mice at 15 weeks (*p* < 0.05, Wilcoxon’s rank sum test) (Fig 3B). In iPLA_2_β-KO mice at 100 weeks, we observed about 70% decrease in DAT immunoreactivity compared with that of WT mice at 100 weeks and iPLA_2_β-KO mice at 56 weeks; this difference was statistically significant (*p* < 0.05, Wilcoxon’s rank sum test) (Fig 3B).

Also by Western blotting, the expression levels of DAT were significantly decreased in the striatum of iPLA_2_β-KO mice at 100 weeks compared with age-matched WT mice (p < 0.05, Wilcoxon's rank sum test, S1 Fig), and to a lesser extent, the expression levels of TH also showed decreases in the striatum of iPLA_2_β-KO mice relative to those of WT mice with statistical significance (p < 0.05, Wilcoxon's rank sum test, S1 Fig).

Moreover, we examined quantitatively the optical densities of VMAT2-immunopositive nerve terminals in the striatum of iPLA_2_β-KO mice at 56 weeks. As shown in S2 Fig, there was a statistically significant decrease in the optical densities of VMAT2 in the striatum of iPLA_2_β-KO mice at 56 weeks compared with that of WT mice at 56 weeks (p < 0.05, Wilcoxon’s rank sum test).

### Progressive Increase in TH-Positive Round Structures in the Striatum of iPLA_2_β-KO Mice

**Fig 4:**
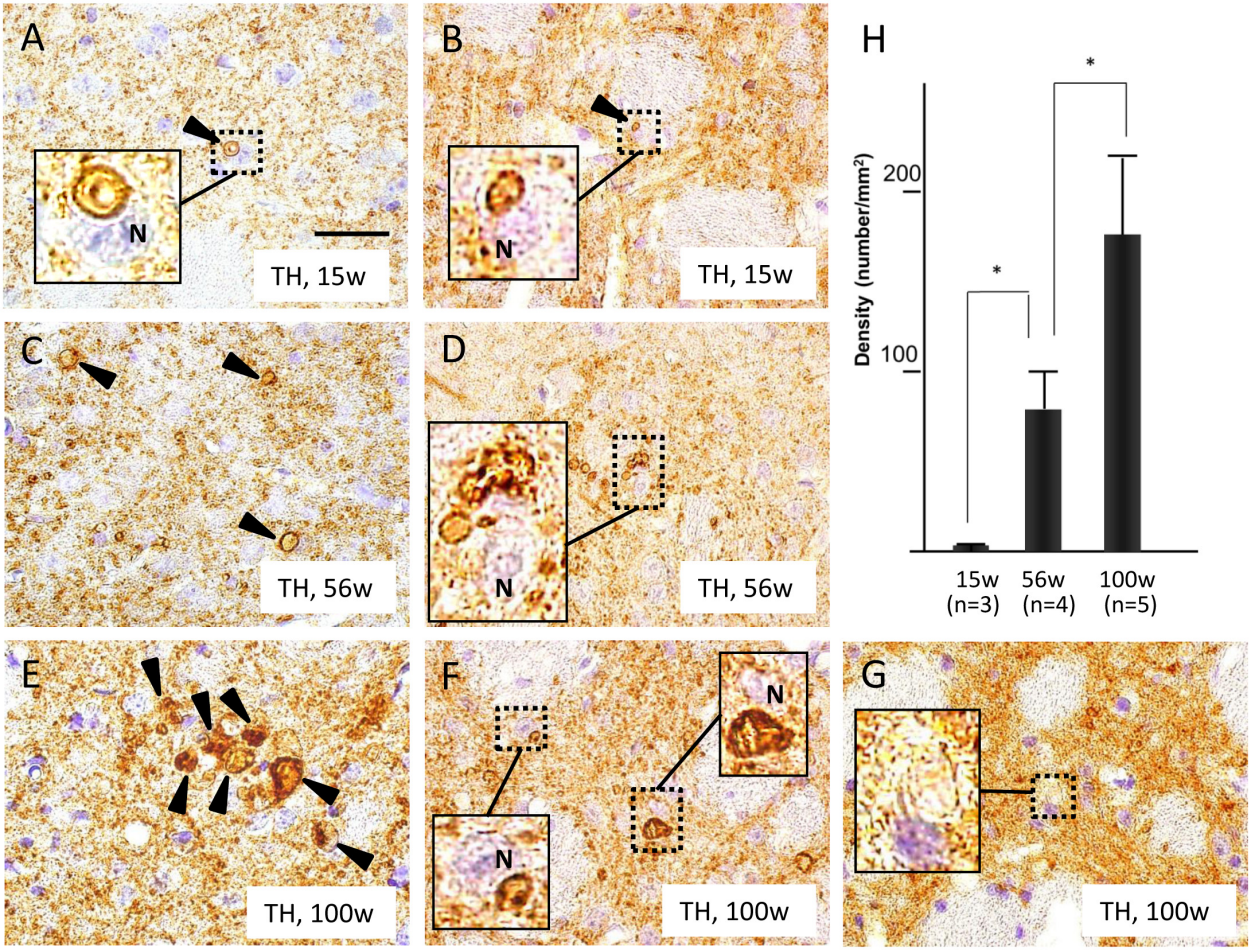
Progressive increase in TH-positive round structures in the striatum of iPLA_2_β-KO mice. (A–G): Representative photographs of the striatum immunostained with TH in iPLA_2_β-KO mice at 15 weeks (A, B), 56 weeks (C, D), and 100 weeks (E, F, G). Insets in (A), (B), (D), (F), and (G) are high magnifications of the dotted squares in their respective panel. Scale bar in (A) represents 25 μm in all panels (A-G). (H): Histogram showing the density (number/mm^2^) of TH-positive round structures in mice at the three ages. Data are presented as the mean ± standard deviation, and symbols indicate significant differences between groups (*p < 0.01, Wilcoxon’s rank sum test).(A, B): Several round, TH-positive structures similar in shape to a vacuole (arrowhead in A) or spheroid (arrowhead in B) were seen in the striatum, contacting striatal neurons (N) as shown in the insets. (C, D): At 56 weeks, the round, TH-positive structures (arrowheads in C) increased in number and size compared to those at 15 weeks (A, B). A striatal neuron (N) is surrounded by several TH-positive structures as shown in the inset (D). (E, F): At 100 weeks, a further increase in the number and size of TH-positive structures is seen (arrowheads in E) compared to those at 56 weeks (C, D). Some of the large, strongly TH-positive structures contact striatal neurons (N) (insets in F). (G): A few TH-negative spheroids (inset) were also seen in the striatum of iPLA_2_β-KO mice. (H): The density of round, TH-positive structures was very low at 15 weeks (n = 3), second highest at 56 weeks (n = 4), and highest at 100 weeks (n = 5), and these differences were statistically significant (*p < 0.01).

In iPLA_2_β-KO mice at 15 weeks, neuropils of the striatum had a few small, round structures similar in appearance to vacuoles (Fig 4A) and spheroids (Fig 4B) that were strongly immunopositive for TH. These structures were not observed in WT mice at 15 weeks (Fig 1B). In iPLA_2_β-KO mice at 56 weeks, these TH-positive structures in neuropils of the striatum increased in size and number (Fig 4C and 4D) compared with those of iPLA_2_β-KO mice at 15 weeks (Fig 4A and 4B). In iPLA_2_β-KO mice at 100 weeks, there were many round structures that were strongly immunopositive for TH. These structures were larger than those observed in iPLA_2_β-KO mice at 56 weeks (Fig 4C and 4D) in neuropils of the striatum (Fig 4E and 4F). A few spheroids negative for TH were also observed (Fig 4G). As shown in Fig 4A, 4B, 4D and 4F, it was suggested that some of the TH-positive structures came in contact with cell bodies of the striatal neurons. In statistical analysis, the number of TH-positive round structures was very small at 15 weeks (n = 3), second largest at 56 weeks (n = 4), and largest at 100 weeks (n = 5), and these differences were statistically significant (*p* < 0.01, Wilcoxon rank sum test) (Fig 4H).

**Fig 5:**
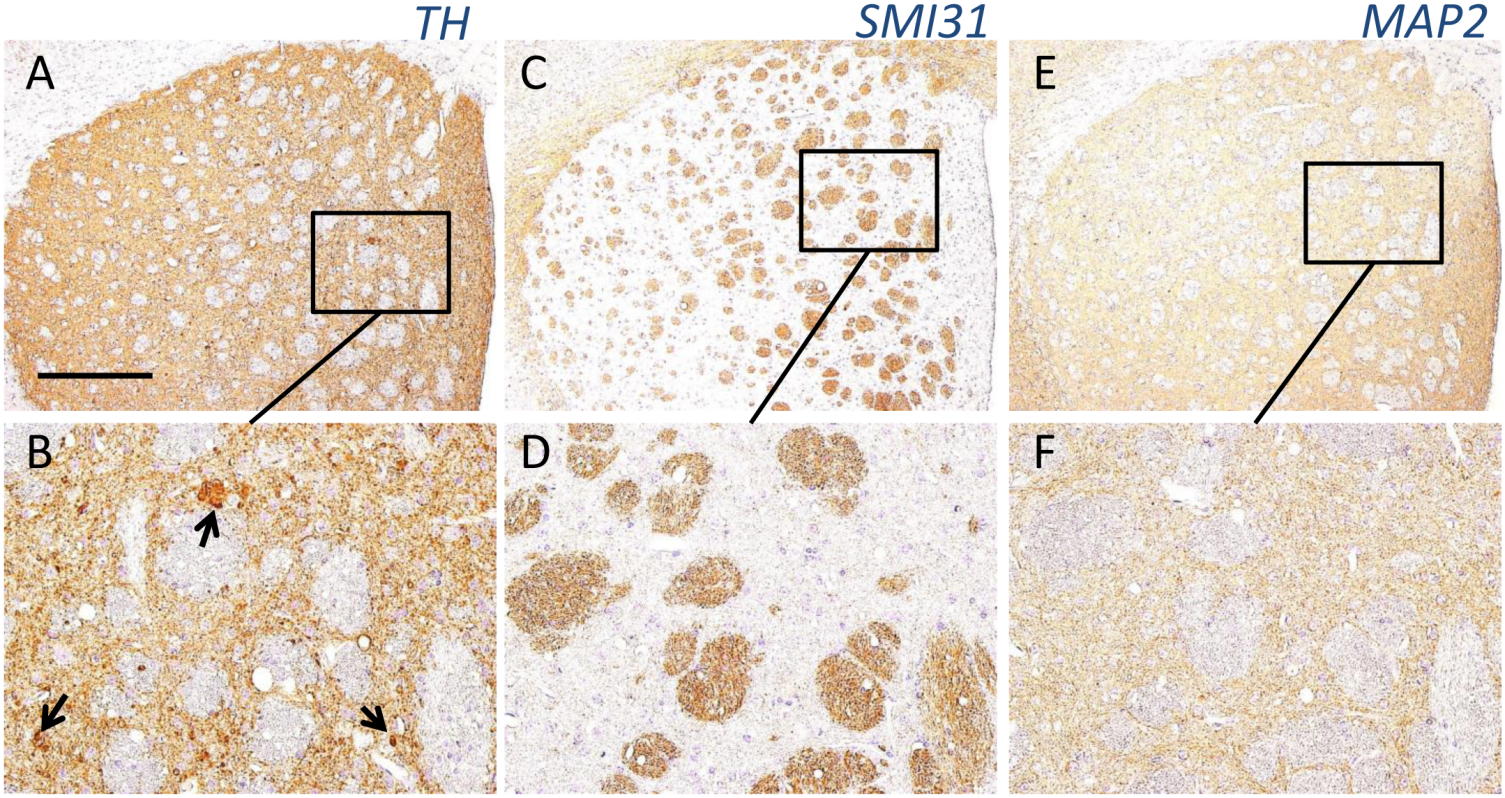
Immunohistochemical analyses of TH, SMI31, and MAP2 in the striatum iPLA_2_β-KO mice at 100 weeks. (A), (C), and (E) are serial sections. (B), (D), and (F) are high magnifications of squares in (A), (C), and (E), respectively. Many TH-positive structures can be seen in the striatum of iPLA_2_β-KO mice at 100 weeks (A, arrows in B), which were mostly negative for SMI31 (C, D) and MAP2 (E, F). Scale bar in (A) represents 200 μm in (A), (C), and (E), and 50 μm in (B), (D), and (F).

**Fig 6:**
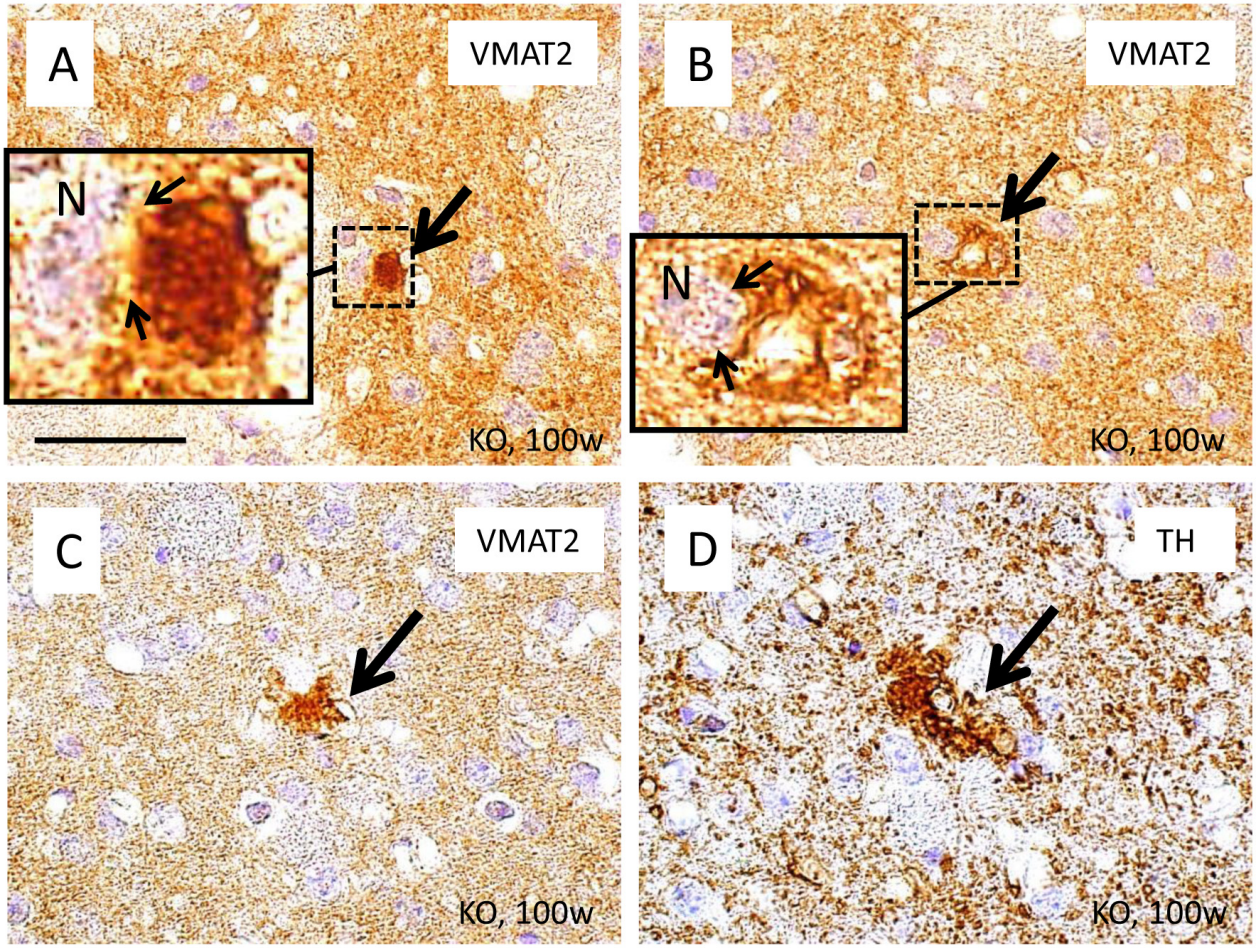
Immunohistochemical analyses of VMAT2 in the striatum of iPLA_2_β-KO mice at 100 weeks. (A, B): Abnormal structures strongly positive for VMAT2 were also seen in KO mice at 100 weeks (large arrows in A and B); these structures contact striatal neurons (N) (small arrows in insets in A and B). In serial sections, an abnormal structure positive for VMAT2 (an arrow in C) is also immunopositive for TH (arrow in D). Scale bar in (A) represents 25 μm in all panels (A-D).

Several TH-positive structures were observed to contact striatal neurons during the pre-clinical stage (Fig 4A and 4B) and to increase with age (Fig 4D and 4F). In contrast, serial sections revealed no such structures positive for phosphorylated neurofilament (SMI31) or MAP2 in the striatum of iPLA_2_β-KO mice, even at the late clinical stage (Fig 5). A few VMAT2-positive, abnormal structures were observed in close proximity to striatal neurons in the oldest iPLA_2_β-KO mice (Fig 6A and 6B). In serial sections, some of the round structures and vacuoles strongly positive for TH (Fig 6D) were also immunopositive for VMAT2 (Fig 6C) in the striatum of iPLA_2_β-KO mice at 100 weeks.

**Fig 7:**
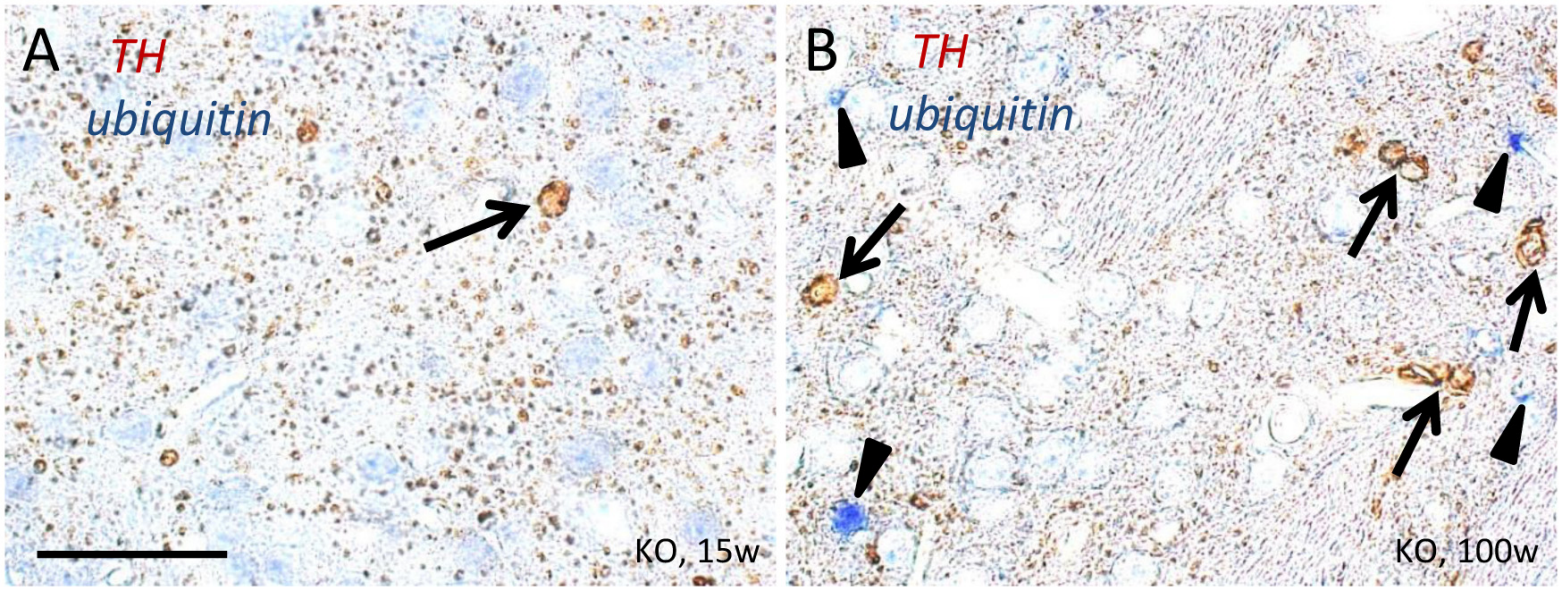
Double immunohistochemistry to detect ubiquitin and TH in the striatum of iPLA_2_β-KO mice. (A, B): Double immunostaining of TH (brown) and ubiquitin (blue) in the striatum of iPLA_2_β-KO mice at 15 weeks (A) and 100 weeks (B). (A): At 15 weeks, a few round, TH-positive structures were seen (brown, an arrow), while blue staining was not found in this view (immunohistochemistry for ubiquitin is negative). (B): At 100 weeks, several ubiquitin-positive structures were found (blue staining, arrowheads), but these were distinct from the round, TH-positive structures (brown staining, arrows). Scale bar in (A) represents 25 μm in (A) and (B).

Accumulations of ubiquitinated proteins were observed in the striatum of iPLA_2_β-KO mice at the early clinical stage and increased with age (Fig 7) as reported previously [9, 10]. At age 15 weeks, a few small, round, TH-positive structures were seen in iPLA_2_β-KO mice, but they were negative for ubiquitin in double immunohistochemical analysis (Fig 7A). Although the number of TH-positive round structures had increased at 100 weeks, all were negative for ubiquitin (Fig 7B).

### Examination of Dopaminergic Nerve Terminals in the Amygdala

**Fig 8:**
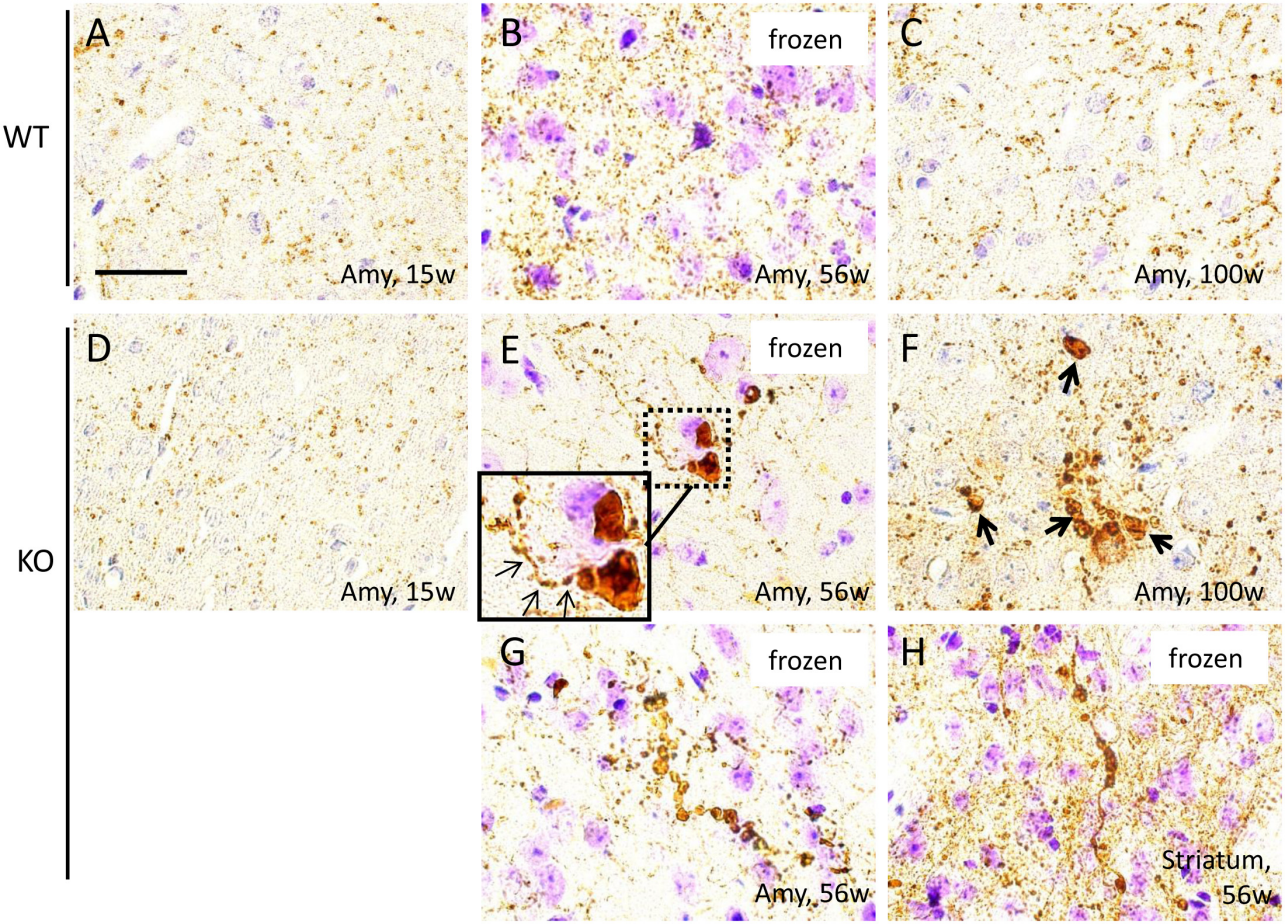
Immunohistochemical analysis of TH in the amygdala of WT control mice and iPLA_2_β-KO mice. Representative photographs of the amygdala immunostained for TH in WT mice at 15 weeks (A), 56 weeks (B), and 100 weeks (C) and iPLA_2_β-KO mice at 15 weeks (D), 56 weeks (E, G), and 100 weeks (F). A section immunostained with TH in the striatum of iPLA_2_β-KO mice at 56 weeks is also shown (H). (B), (E), (G), and (H) are frozen sections. The inset in (E) is a high magnification of the dotted square. (A–C): Distal regions of the axons of dopaminergic neurons are positive for TH in the amygdala of WT mice. (D): In iPLA_2_β-KO mice at 15 weeks, TH-positive nerve fibers similar to those of WT mice were observed (A-C). (E, G): In iPLA_2_β-KO mice at 56 weeks, round structures positive for TH are seen. Some are adjacent to TH-positive nerve fibers (small arrows in E). The round, TH-positive structures lie in a row like a string of beads (G). (F): Many round, TH-positive structures were seen in iPLA_2_β-KO mice at 100 weeks (arrows in F). (H): Beaded, round TH-positive structures were also seen in the striatum of iPLA_2_β-KO mice at 56 weeks. Scale bar in (A) represents 25 μm in all panels (A-H).

Dopaminergic neurons in the ventral tegmentum are known to send projections to the amygdala. We observed that nerve fibers in the amygdala were positive for TH in mice of all ages (Fig 8A, 8B and 8C). The staining patterns of TH immunohistochemistry in the amygdala were similar in iPLA_2_β-KO mice at 15 weeks (Fig 8D) and WT mice (Fig 8A–8C). On the other hand, round structures strongly positive for TH were found in iPLA_2_β-KO mice at 56 weeks (Fig 8E and 8G), and these structures increased in number and size in iPLA_2_β-KO mice at 100 weeks (Fig 8F). Some of the TH-positive fibers lead to round structures that stained strongly for TH and resemble axon terminals (Fig 8E). Moreover, beaded, round structures strongly immunopositive for TH were observed in the amygdala (Fig 8G) as well as the striatum (Fig 8H) of iPLA_2_β-KO mice at 56 weeks.

## Discussion

In the present study, we observed decreases in TH and DAT in the striatum starting in the early clinical stage in iPLA_2_β-KO mice. Dopaminergic neurons in the substantia nigra (SN) were not depleted at the same age as in our previous report [22]. We also observed an age-dependent increase in strongly TH-positive structures, likely deformed axons, in the striatum of iPLA_2_β-KO mice. Our results suggest that a deficiency of iPLA_2_β leads to the degeneration of distal regions of axons of nigrostriatal dopamine neurons. Decreased DAT expression indicates downregulation of presynaptic dopamine reuptake, and these changes in expression are compensatory and are observed in the brains of patients with Parkinson’s disease (PD) [20, 21].

Numerous reports have shown that the expression levels of TH and DAT were reliable estimates of dopaminergic nerve terminal density in the striatum in both patients with PD [20, 21] and animal models [23, 24, 25]. The damage to dopaminergic neurons in the SN is thought to cause a loss of striatal nerve terminals, producing corresponding changes in striatal levels of these presynaptic markers. In our previous study, we observed no significant difference in the number of TH-positive dopaminergic neurons in the SNpc between WT and iPLA_2_β-KO mice at 56 weeks [22]. In the present study, we observed a decrease in the densities of TH- and DAT-positive fibers in the striatum of iPLA_2_β-KO mice at 56 weeks compared with age-matched WT mice, suggesting that the degeneration of dopaminergic neurons occurs in the distal regions of axons in iPLA_2_β-KO mice. Moreover, we showed that the densities of VMAT2-positive fibers in the striatum of iPLA_2_β-KO mice at 56 weeks were decreased in comparison with those of age-matched WT mice. These results suggest a loss of axon terminals in the striatum of iPLA_2_β-KO mice at 56 weeks and that the decreased expression levels of TH and DAT would be due to a consequence of terminal loss.

Quantitative analysis revealed that the decrease in DAT-positive fibers was greater than that of TH-positive fibers in the striatum of iPLA_2_β-KO mice. DAT is an integral membrane protein that removes dopamine from the synaptic cleft and deposits it inside surrounding cells, thus terminating the neurotransmitter signal [19, 20, 21]. A possible explanation for our observations is that the downregulation of DAT, which prevents this means of dopamine signal termination, may be a compensatory mechanism to maintain optimal synaptic dopaminergic transmission [19, 21]. In addition, the downregulation of DAT suggests the presence of synaptic dysfunction in iPLA_2_β-KO mice. A significant decrease in DAT was also observed in the striatum of in humans with *PLA2G6* mutations using positron emission tomography (PET) [14].

The appearance of symptoms in animal models of PD is thought to require a 70%–80% loss of striatal terminals and a 50%–60% loss of dopaminergic neurons [24]. Motor deficits in iPLA_2_β-KO mice are not caused by a decrease in the number of dopaminergic neurons but might instead be induced by axonal and synaptic dysfunction in the striatum. Axonal degeneration was also observed in the spinal cords and peripheral nerves from the pre-clinical stage of iPLA_2_β-KO mice [10, 11], which could induce motor symptoms.

We also observed the emergence of abnormal, round structures strongly immunopositive for TH in neuropils of the striatum of iPLA_2_β-KO mice that increased in number and size with age. These structures looked like axonal spheroids, and some of them were lined up in a row, suggesting that they might be deformed axons of dopaminergic neurons. Although we found the increased immunoreactivities for TH in spheroids and other forms of degenerated axon terminals, the meanings of these observations remain unclear. Some VMAT2-positive structures were also observed in the striatum of older iPLA_2_β-KO mice. Because VMAT2 is essential for the release of neurotransmitters into the synaptic cleft by presynaptic neurons [19, 20, 21], VMAT2 upregulation might be a compensatory mechanism for synaptic dysfunction, such as impaired dopamine release. These structures were negative for phosphorylated neurofilament (SMI31). This observation could be explained by the focal loss of neurofilaments in the deformed axons, as shown in the ultrastructural analysis in our previous report [11]. Another plausible explanation is that some of the round structures were deformed axon terminals where neurofilaments were absent [26]. Some of the round structures were close to the medium spiny neurons in the striatum, suggesting deformed axon terminals.

At the same time, the round, TH-positive structures were negative for ubiquitin. In the striatum of iPLA_2_β-KO mice, accumulation of ubiquitinated proteins was observed in the pre-clinical stage and increased with age, implying an association with neurodegeneration [9, 10]. In addition, extensive ubiquitin-positive degenerating neurites have been observed in the brains of patients with PD [27]. Although we demonstrated that deformed axons with high TH expression in iPLA_2_β-KO mice showed no accumulation of ubiquitinated proteins, the significance of these findings remains unclear. Moreover, most of the round structures in the striatum were negative for 4-hydroxy-2-nonenal, an oxidized secondary product formed when organic lipids consisting of polyunsaturated fatty acids are exposed to oxidative stress, as reported previously [22]. This product might be identical with TH-positive structures without accumulation of peroxidized lipids.

Degeneration of dopamine nerve terminals and synaptic dysfunction in the striatum were recently reported in the pathogenesis of PD [28, 29]. PD-linked proteins, such as α-synuclein, parkin, and leucine-rich repeat kinase 2 (LRRK2), localize to synaptic membranes and are associated with membrane trafficking [30]. Our results in iPLA_2_β-KO mice suggest that degeneration of striatal dopamine nerve terminals might be a common pathogenic mechanism in sporadic and familial PD.

In conclusion, iPLA_2_β-KO mice showed age-dependent degeneration of the distal region of axons of nigrostriatal dopamine neurons. This pathological change likely results in synaptic dysfunction. The iPLA_2_β-KO mouse is an ideal animal model for studying Parkinsonian symptoms caused by impairment of the dopaminergic nervous system, as seen in sporadic PD as well as PARK14. Further investigations of this animal model should provide useful information regarding the pathogenesis of neurodegenerative diseases with Parkinsonism.

## Supporting Information

S1 Fig Western blotting analyses of TH and DAT in the striatum of iPLA_2_β-KO mice at 100 weeks. (A) Western blotting was applied to detect the expressions of TH (molecular weight: 60kDa) and DAT (molecular weight: 88kDa) in the striatum of iPLA_2_β-KO mice (n = 3) and WT mice (n = 3) at 100 weeks. (B) Statistical analysis. Data are presented as the ratio of TH or DAT to GAPDH (WT mice = 1.0). Each bar represents the mean ± SD. *p < 0.05, Wilcoxon's rank-sum test.
(TIF)

S2 Fig Quantitative analysis of optical densities of VMAT2 in the striatum. (A, B); Representative photographs of the striatum immunostained with VMAT2 in WT mice at 56 weeks (A) and iPLA_2_β-KO mice at 56 weeks (B) are shown. Scale bar in (A) represents 25 μm in (A) and (B). (C) Histograms show quantitative analysis of optical densities of VMAT2 immunostaining in the striatum. WT mice, gray bars; KO mice, black bars. Data are presented as the mean ± standard deviation. The number (n) of animals examined is indicated in each histogram. Vertical axis shows percent density relative to WT mice. Symbols indicate statistically significant differences; *p < 0.05 vs. WT mice (Wilcoxon’s rank sum test).
(TIF)
