# Supplementary figures and images for: Progressive Axonal Degeneration of Nigrostriatal Dopaminergic Neurons in Calcium-Independent Phospholipase A2β Knockout Mice
Source: PLoS One. 2016 Apr 14;11(4):e0153789. doi: 10.1371/journal.pone.0153789 (PMC4831782; doi:10.1371/journal.pone.0153789)

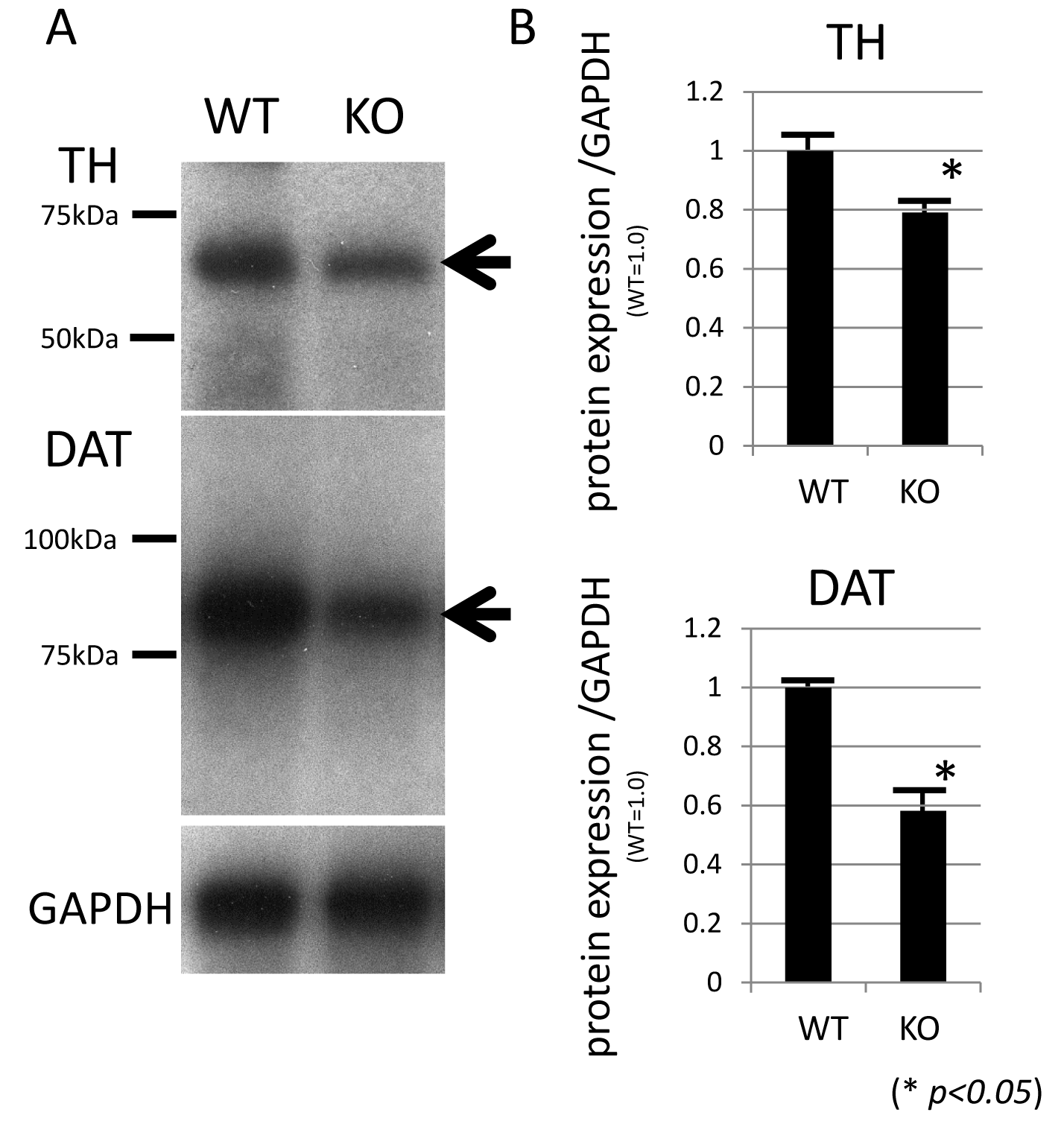

Supplement: S1 Fig — (A) Western blotting was applied to detect the expressions of TH (molecular weight: 60kDa) and DAT (molecular weight: 88kDa) in the striatum of iPLA2β-KO mice (n = 3) and WT mice (n = 3) at 100 weeks. (B) Statistical analysis. Data are presented as the ratio of TH or DAT to GAPDH (WT mice = 1.0). Each bar represents the mean ± SD. *p < 0.05, Wilcoxon's rank-sum test. (TIF) [file pone.0153789.s001.tif]

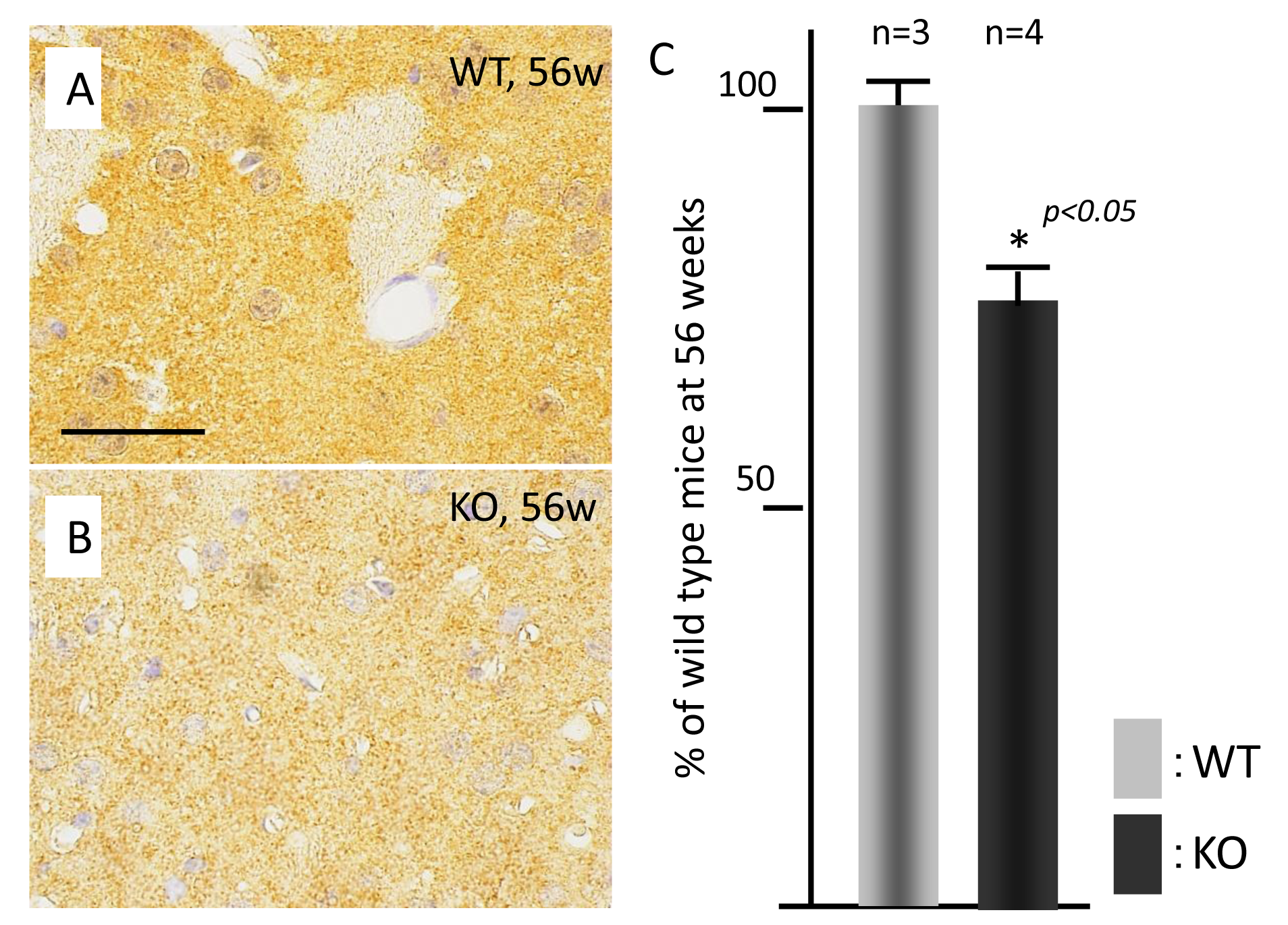

Supplement: S2 Fig — (A, B); Representative photographs of the striatum immunostained with VMAT2 in WT mice at 56 weeks (A) and iPLA2β-KO mice at 56 weeks (B) are shown. Scale bar in (A) represents 25 μm in (A) and (B). (C) Histograms show quantitative analysis of optical densities of VMAT2 immunostaining in the striatum. WT mice, gray bars; KO mice, black bars. Data are presented as the mean ± standard deviation. The number (n) of animals examined is indicated in each histogram. Vertical axis shows percent density relative to WT mice. Symbols indicate statistically significant differences; *p < 0.05 vs. WT mice (Wilcoxon’s rank sum test). (TIF) [file pone.0153789.s002.tif]
